# Genomic analysis of the emergence of drug-resistant strains of *Mycobacterium tuberculosis* in the Middle East

**DOI:** 10.1038/s41598-019-41162-9

**Published:** 2019-03-14

**Authors:** Essam J. Alyamani, Sarah A. Marcus, Sarah M. Ramirez-Busby, Chungyi Hansen, Julien Rashid, Amani El-kholy, Daniel Spalink, Faramarz Valafar, Hussein A. Almehdar, Asif A. Jiman-Fatani, Mohamed A. Khiyami, Adel M. Talaat

**Affiliations:** 1 National Center for Biotechnology, King Abdulaziz City for Science and Technology, Riyadh, Saudi Arabia; 2 Department of Pathobiological Sciences, University of Wisconsin-Madison, Madison, WI USA; 3 Laboratory for Pathogenesis of Clinical Drug Resistance and Persistence, Biomedical Informatics Research Center, San Diego State University, San Diego, CA USA; 4 Clinical Pathology Department, Faculty of Medicine Cairo University, Cairo, Egypt; 5 Department of Ecosystem Science and Management, Texas A&M University, College Station, TX USA; 6 Department of Medical Microbiology and Parasitology, Faculty of Medicine, King Abdulaziz University, Jeddah, Saudi Arabia; 7 Department of Biology, Faculty of Science, King Abdulaziz University, Jeddah, Saudi Arabia

**Keywords:** Microbiology, Pathogens

## Abstract

Tuberculosis (TB) represents a significant challenge to public health authorities, especially with the emergence of drug-resistant (DR) and multidrug-resistant (MDR) isolates of *Mycobacterium tuberculosis*. We sought to examine the genomic variations among recently isolated strains of *M. tuberculosis* in two closely related countries with different population demography in the Middle East. Clinical isolates of *M. tuberculosis* from both Egypt and Saudi Arabia were subjected to phenotypic and genotypic analysis on gene and genome-wide levels. Isolates with MDR phenotypes were highly prevalent in Egypt (up to 35%) despite its relatively stable population structure (sympatric pattern). MDR-TB isolates were not identified in the isolates from Saudi Arabia despite its active guest worker program (allopatric pattern). However, tuberculosis isolates from Saudi Arabia, where lineage 4 was more prevalent (>65%), showed more diversity than isolates from Egypt, where lineage 3 was the most prevalent (>75%). Phylogenetic and molecular dating analyses indicated that lineages from Egypt were recently diverged (~78 years), whereas those from Saudi Arabia were diverged by over 200 years. Interestingly, DR isolates did not appear to cluster together or spread more widely than drug-sensitive isolates, suggesting poor treatment as the main cause for emergence of drug resistance rather than more virulence or more capacity to persist.

## Introduction

Tuberculosis (TB) remains a public health threat^1^, especially in developing countries where infection rates are increasing and diagnosis is a significant challenge^1^. Genotypic variations among clinical isolates of *Mycobacterium tuberculosis*, the causative agent of TB, are associated with specific phenotypes (e.g., resistance to autophagy)^2^, which highlights the importance of profiling the population structure of *M. tuberculosis* in all communities. Worldwide, the incidence of TB has reached an alarming level, especially in sub-Saharan Africa and South-East Asia (300+ per 100,000)^3^. With ~10.0 million new TB cases in 2014 alone, understanding the basis for disease transmission and screening for drug-resistant strains are essential for an effective control strategy^4^. In the Middle East, drug-resistant isolates of *M. tuberculosis* are of significant importance and require further scrutiny. This is particularly important in Saudi Arabia^5,6^ and Egypt^7,8^ where disease transmission dynamics are not affected by high levels of infection with human immunodeficiency virus (HIV) found in sub-Saharan Africa^9^. Migrant workers from countries with high prevalence of tuberculosis, such as South-East Asian countries, are often implicated in TB transmission beyond regional borders and require close monitoring by the public health authority^10^. This is the case in the Kingdom of Saudi Arabia, especially among foreign workers^11^, where TB constitutes a significant health problem. Recent reports have indicated the presence of multiple lineages of *M. tuberculosis* in Saudi Arabia, a country with an active guest workers program^12^. Annual visitors for religious rituals in Saudi Arabia may also contribute to the transmission of TB. In Egypt, another Middle Eastern country with a stable population where guest workers are rare, TB is still considered a significant problem^13^. Until now, the population structure of *M. tuberculosis* in those countries with historic ties to global trade remain elusive. In this study, we perform comparative analyses of whole genome sequences (WGS) to profile the dynamics of TB transmission in a relatively stable community (e.g., Egypt) compared to those undergoing significant social changes (e.g., Saudi Arabia).

Earlier reports provided clear evidence for the emergence of single- and multidrug- resistant tuberculosis (MDR-TB) in the Middle East. More importantly, they indicated the drastic variability in prevalence of single drug-resistant and MDR-TB (ranging from 3–50%)^8,14–16^, most likely related to lack of adherence to TB drug treatment. Earlier studies used growth-based phenotyping methods^8,14^, while more recent studies have employed a combination of genotypic approaches such as PCR and other targeted sequencing approaches to detect resistance-conferring mutations^5,17^. Information about the predominance of *M. tuberculosis* lineages in the Middle East has only been rudimentary and scarce, especially on a genome-wide level, until recently^18^. Analysis of a large number of *M. tuberculosis* isolates collected from different countries around the globe identified resistance to isoniazid (*katG* mutation) as a precursor for the emergence of MDR-TB and other more complex drug resistance profiles^19^. Unfortunately, there were no isolates included in this study from the Middle East, despite the inclusion of 8,316 other isolates^19^. More recently, a total of 76 isolates were analyzed using WGS from Saudi Arabia^18^. Studies have documented increased risks of TB incidence among “Hajj” pilgrims in Saudi Arabia, due to mass-gatherings and a large concentration of people from all over the world^20,21^. Some of these countries are described as countries with high-incidence of TB^19^. Furthermore, an increase drug-resistant (DR) strains among MDR-TB particularly to aminoglycoside and fluoroquinolone have been documented in Saudi Arabia recently^5^. The current study represents the first attempt to utilize WGS of clinical *M. tuberculosis* isolates from both Egypt and Saudi Arabia to profile the population structure of this important pathogen considering the patient’s country of origin for each isolate. Findings from our analysis suggest the significance of the problem of DR and MDR among *M. tuberculosis* isolates in the Middle East, particularly in stable communities (up to 35%), is actually much higher than identified before on a global level (18%)^19^ with little influence of migrant workers on disease transmission dynamics.

## Results

### Predominant lineages of *M. tuberculosis* in the Middle East

To characterize *M. tuberculosis* in the Middle East, we analyzed the genomes of archived isolates from sputum samples of 69 patients at the King Abdulaziz University Hospital (KAUH) in Jeddah, Saudi Arabia, that frequented the chest ward between January 2012 and March 2013. In addition, we analyzed the genomes of archived isolates from 60 patients at Cairo University, Cairo, Egypt, that frequented the university chest clinic during the same period. Of the total 129 samples, 95 isolates (61 from Saudi Arabia and 34 from Egypt) were chosen for genomic analysis based on successful culturing and ability to extract high-quality genomic DNA. For Egypt, all isolates were from Egyptian natives, while isolates from Saudi Arabia were collected from patients native to a range of countries in Africa (Chad, Somalia), the Arabian Peninsula (Saudi Arabia, Yemen), and Asia (Afghanistan, India, Myanmar, Indonesia, the Philippines). Patients ranged in age from 1 year to 76 years old with nearly half of them falling between ages 21 and 40. More demographic information of the patients is presented in Table 1. The results of the examination of sputum samples for the presence of *M. tuberculosis* and the drug sensitivity tests are compiled in Table 2.

**Table 1:** Patient demographics information for both Egypt and Saudi Arabia.

	Egypt		Saudi Arabia	
	Count	Percent*	Count	Percent*
**Age**				
<11	0	0%	6	10%
11–20	1	2.90%	9	15%
21–40	18	52.90%	30	49%
41–60	10	29.40%	9	15%
>60	5	14.70%	7	11%
**Gender**				
Male	29	85.30%	27	44%
Female	5	14.70%	34	56%
**Nationality**				
Egyptian	34	100%	0	0
Saudi			21	34%
Somali			15	25%
Yemeni			7	11%
Chadian			4	7%
Pakistani			3	5%
Afghan			2	3%
Ethiopian			2	3%
Filipino			2	3%
Indonesian			2	3%
Indian			1	2%
Malian			1	2%
Burmese			1	2%
**City of Residence**				
Greater Cairo, Egypt	23	67.60%		
Fayoum, Egypt	11	32.40%		
Asir, Saudi Arabia			1	2%
Jeddah, Saudi Arabia			46	75%
Makkah, Saudi Arabia			3	5%
Unknown city within Saudi Arabia			11	18%
**Total isolates**	**34**	**100%**	**61**	**100%**

*Percentages are calculated based on the total number of isolates (N = 95) that were cultured from sputum samples received from both Egypt (N = 34) and Saudi Arabia (N = 61).

**Table 2:** Drug resistance profile of analyzed *M. tuberculosis* isolates from Egypt and Saudi Arabia.

Acid Fast Bacilli (AFB)*	Egypt		Saudi Arabia	
	Number**	Percent**	Number**	Percent**
No AFB seen	1	2.90%	15	25%
1–9 AFB	11	32.40%	15	25%
+	15	44.10%	10	16%
+ +	7	20.60%	7	11%
+++			10	16%
Inconclusive			4	7%
Resistance***				
Isoniazid	12 (2)	35.3% (5.9%)	1	1.6%
Ethambutol	13 (0)	34.2% (0)	3 (0)	4.9% (0%)
Pyrazinamide	0	0	3	5%
Streptomycin	3	8.80%	4 (3)	6.5% (5%)
Rifampicin	13 (4)	34.2% (11.8%)	0	0%
Pan-sensitive	2 (22)	5.9% (64.7%)	52 (55)	85.2 (90.1%)
Double DR (ET, S)	0	0.00%	2 (0)	3.3% (0%)
MDR (INH, ET, R)	12 (2)	35.3% (5.9%)	0	0
MDR (INH, S, R, PZ, ET)	2	5.90%	0	0
**Total isolates**	**34**		**61**	

*Scores represent the level of acid fast bacilli (AFB) in each sample.

>10 AFB/field in 20+ fields:+++.

1–10 AFB/field in 50+ fields:++.

10–99 AFB/ 100 fields: +.

1–9 AFB/100 fields: 1–9.

0 AFB/ 300 fields: No AFB seen.

**Where they differ from UW-Madison laboratory testing, Original numbers and percentages reported from clinical laboratories are listed in parenthesis

***S: streptomycin, R: rifampicin, INH: isoniazid, PZ: pyrazinamide, ET: ethambutol, MDR: multi-drug-resistant.

**Figure 1:**
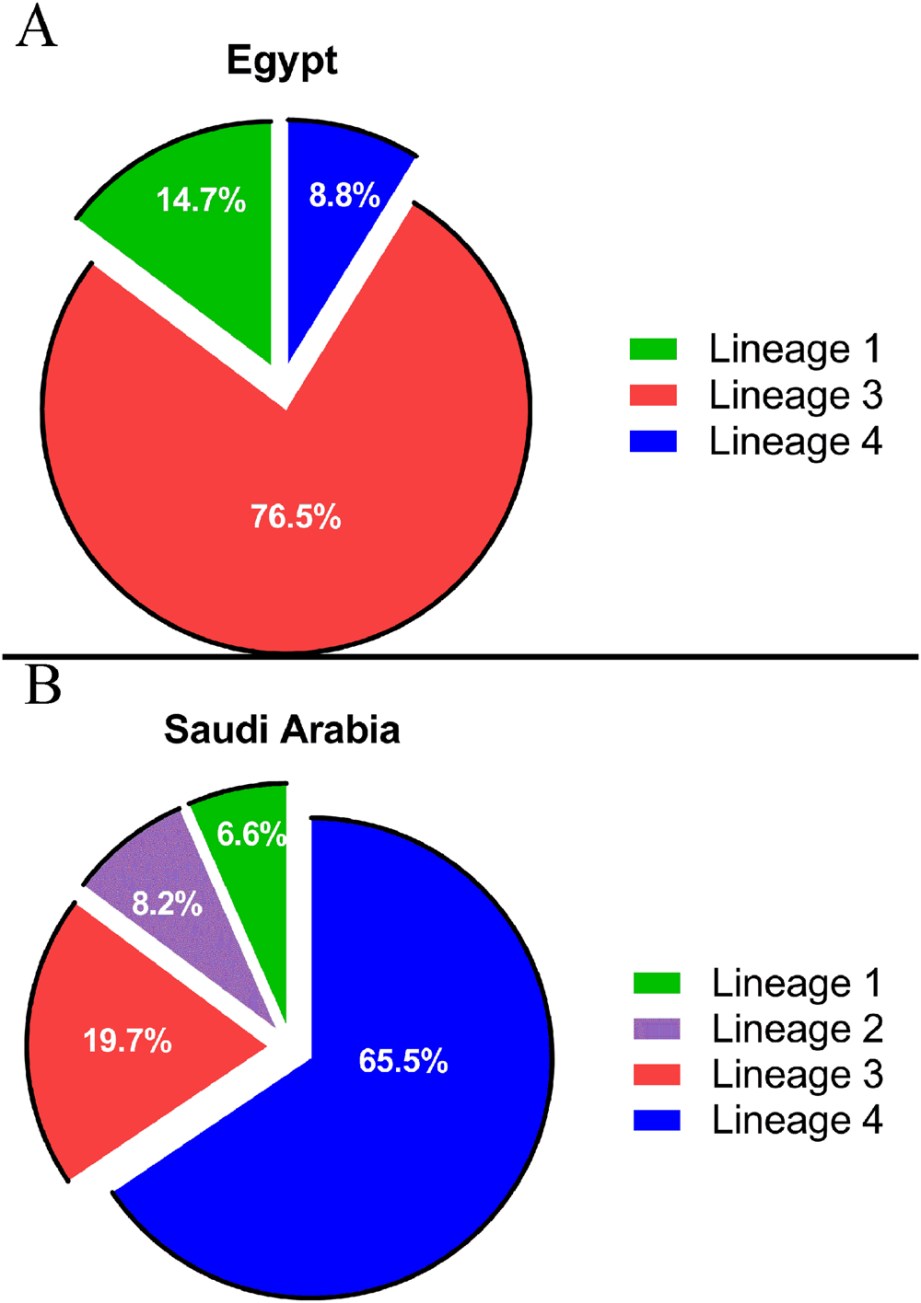
Lineage distribution based on MIRU-VNTR typing for isolates from both (**A**) Egypt and (**B**) Saudi Arabia. If a conflict of lineage assignment occurred, PCR tests were performed to assign the appropriate lineage.

To analyze the population structure of *M. tuberculosis* isolates, we employed a protocol based on 12-locus MIRU-VNTR typing^22^. Because of the demographic differences between the Egyptian and Saudi Arabian sources of the *M. tuberculosis* isolates under study (Table 1), we hypothesized the presence of different models for disease transmission and spread dynamics. In Egypt, we expected the predominance of a countrywide group of isolates to cluster in a sympatric pattern (isolates from a single geographical region with a stable population) compared to Saudi Arabia with more diversity of *M. tuberculosis* lineages in an allopatric pattern (isolates from multiple, isolated geographical regions with a diverse population)^23^. Out of 25 genotypes identified (out of 34 isolates), the most predominant lineage in Egypt was from the Indian-East African lineage (lineage 3), which was represented in 76.5% of examined isolates (Fig. 1). On the other hand, out of 59 genotypes identified (out of 61 isolates), the Euro-American (lineage 4) was represented in 66% of isolates in Saudi Arabia (Fig. 1), confirming earlier reports^24^. Analysis on the MIRU-VNTR website^25^ further categorized the isolates into sub-lineages summarized in Table 3. Moreover, the lineages of 16 isolates (out of 61 isolates) from Saudi Arabia were predicted differently depending on the algorithm used (TBinsight^26^ vs. MIRU-VNTRplus). To resolve discordance between the two databases, we used PCR of pks15/1 to distinguish between lineages 3 and 4. Interestingly, two isolates from Saudi Arabia, SA34 and SA60, of lineage 4, did not group with any known sub-lineage and appear to be specific to Saudi Arabia. The genomes of these two strains, among others from Egypt and Saudi Arabia, were subjected to further analysis using whole genome sequencing.

**Table 3:** Lineage differences between two databases of KSA and Egypt samples.

	Lineage 1	Lineage 2	Lineage 3	Lineage 4	Others*	Total
KSA TBinsight	4	5	8	44	0	61
KSA MIRU-VNTRplus	3	1	9	41	7	61
Egypt TBinsight	5	0	25	3	0	33
Egypt MIRU-VNTRplus	5	0	21	7	0	33

*Samples not assigned to lineages 1, 2, 3, or 4.

### Sequence variations on a whole genome level

**Figure 2:**
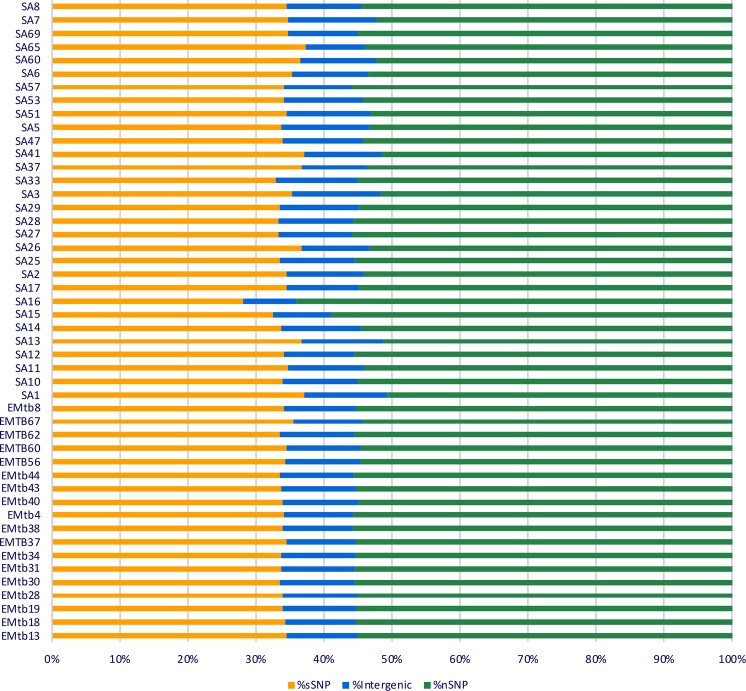
Percentage of synonymous SNPs (sSNP), non-synonymous SNPs (nSNP) and SNPs located in intergenic areas (intergenic). SNPs were included if coverage was more than 20 reads and frequency was ≥50% of the reads.

To gain more insights into diversity among the identified lineages of *M. tuberculosis* from the Middle East, we selected certain isolates for whole genome sequence (WGS) analysis on the Illumina platform. Our selection included isolates with drug resistance phenotypes, representative samples for each MIRU-VNTR cluster (N = 49, 19 from Egypt and 30 from Saudi Arabia), in addition to isolates with unassigned lineages. Sequencing isolate genomes yielded paired-end fragments of 101 bp (~805-fold coverage) or 251 bp (~114-fold coverage), respectively. The total number of reads, number of mapped reads, and coverage data for each genome can be found in Table S1. Whole genome sequences were aligned using bowtie^27^, and SNPS and insertions/deletions (indels) were identified using Varscan2^28^. Several of these SNPs (N = 20), including those associated with drug resistance (N = 10), were confirmed by classical Sanger sequencing (Table S2). Interestingly, the majority of SNPs were nSNPs (Fig. 2) suggesting a rapidly evolving *M. tuberculosis* population in those patients. Additionally, the number of SNPs between all isolates was high (>100 SNPs), an indication of recent infection rather than infection relapse^29^, from a chronic or latent infection.

In another analysis, we compared WGS-based lineage prediction with MIRU-VNTR (TBinsight analysis^26^) results. In this comparison, lineage assignments of 2 isolates (SA33 and SA47) were discordant where WGS moved them from lineage 4 to lineage 3. Among the 16 isolates that gave different lineage results between TB insight and MIRU-VNTR websites, seven were sequenced, and only one agreed with MIRU-VNTR while all others agreed with TBinsight. Moreover, one previous study of *M. tuberculosis* genotypes found in Saudi Arabia discovered 2 strains with novel spoligotypes (703777707770371, 467777377413771), unique to Saudi Arabia. To determine if these spoligotypes were represented in our samples, *in silico* spoligotyping was done for each sequenced sample^30^. None matched the novel spoligotypes identified by Al-Hajoj, *et al*.^24^, a reflection of targeting a different population from the earlier study.

### Transmission dynamics of *M. tuberculosis* isolates within individual countries

**Figure 3:**
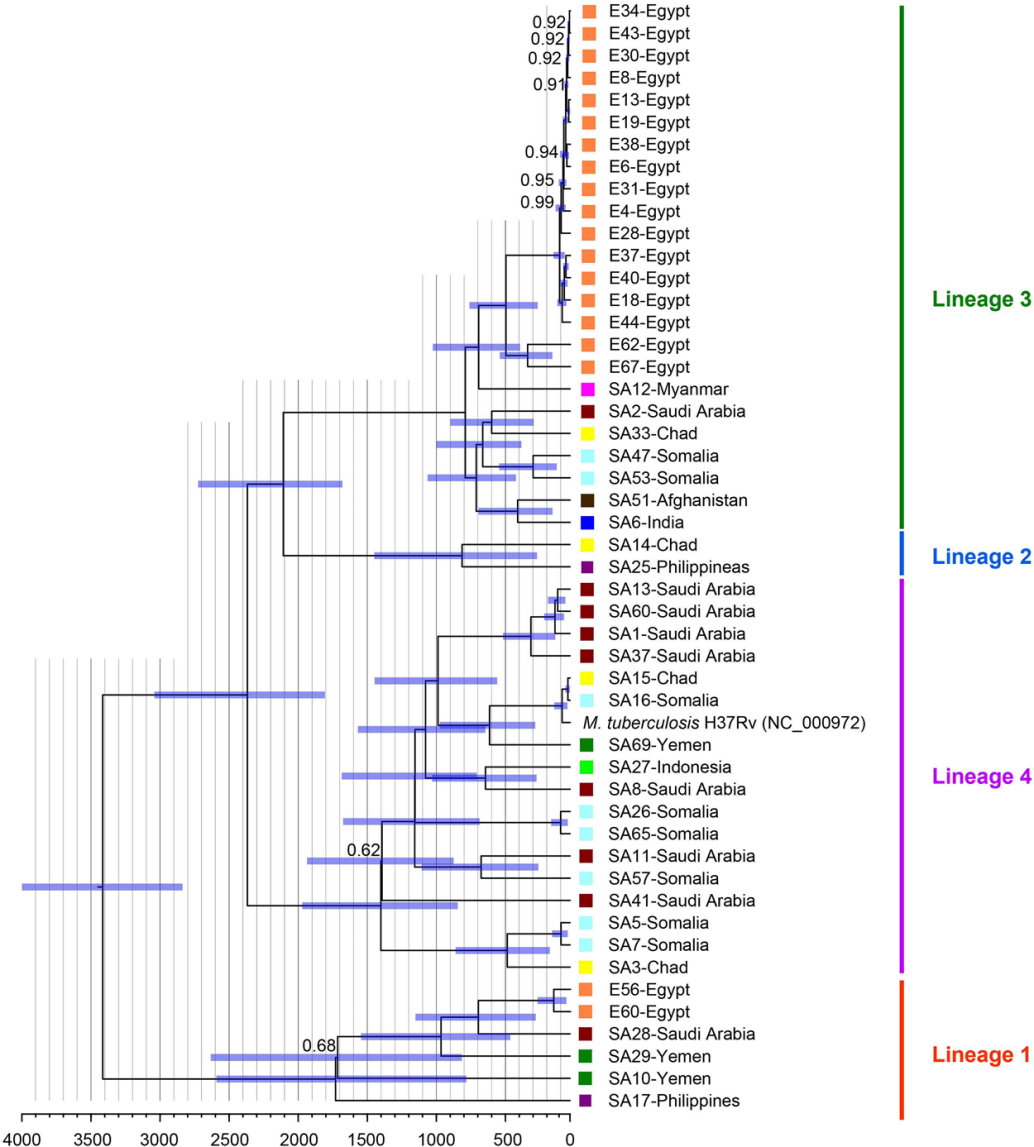
Evolutionary relationships and divergence dates of isolates from Egypt and Saudi Arabia. Divergence dates are in years, with the 95% confidence intervals of node ages represented by blue bars. The topology was recovered with 100% posterior probability, except for those nodes labeled. Color coded boxes at the tips indicate the nationality of the patient from which the sample was isolated and correspond with the color of countries mapped in Fig. 4. SA tip labels refer to samples isolated in Saudi Arabia, whereas E tip labels refer to those isolated in Egypt.

To better understand the nature of *M. tuberculosis* isolate transmission within a population, phylogenetics of isolate variants were analyzed to reconstruct their evolutionary relationship. This phylogenetic analysis was based on the alignment of 17,358 SNPs detected in all 49 sequenced genomes when aligned to the genome of the standard laboratory strain, H37Rv (NC_000962.3). Likelihood ratio tests rejected a molecular clock, so downstream analyses were based on chronogram where rates of evolution were allowed to vary among branches. The resulting tree topology and divergence time estimates among major lineages were consistent with previous estimates^31^, with the most recent common ancestor (MRCA) of all isolates occurring ~3400 years ago. The phylogeny indicates that the vast majority of isolates from Egypt form a single clade in lineage 3, all of which diverged within the past 500 years, and two remaining isolates forming a clade in lineage 1 with an MRCA of ~150 years (Fig. 3). Nearly 80% of isolates from Egypt form a single clade with a crown age of ~78 years.

The isolates from patients in Saudi Arabia were scattered throughout lineages 1–4, with no apparent clustering according to the nationality of the patients. In contrast, isolates from Egypt were significantly more closely related to each other than expected when compared to a null model where nationalities were randomized across the tree (p < 0.01). No other nationalities showed significant phylogenetic clustering. Nevertheless, in some instances isolates from individuals with the same nationality form clades (Fig. 3**)**. For example, isolates from individuals from Somalia form two different clades in lineage 4 and a clade in lineage 3. With few exceptions, divergence dates of closely related isolates from patients in Saudi Arabia were older than 200 years old. Additionally, we found no significant clustering of TB isolates based on patient’s age, or gender (*p* > 0.05), another indication of a dynamic model of disease transmission. Surprisingly, however, was the lack of clustering among isolates from patients sharing the same nationality. This indicates recent spread of *M. tuberculosis* among guest workers, confirming region-specific lineage transmission patterns (sympatric model), even within Saudi Arabia with active guest worker programs, refuting our earlier hypothesis.

### Drug resistance of *M. tuberculosis* isolates

In both Egypt and Saudi Arabia, *M. tuberculosis* isolates were screened for resistance to rifampicin (RMP), isoniazid (INH), ethambutol (EMB), streptomycin (STM), and pyrazinamide (PZA). To further analyze drug resistance among the 49 isolates (from both Egypt and Saudi Arabia) with whole genome sequences, the drug resistance profile of each isolate was re-tested by the group at the University of Wisconsin-Madison against STM, RMP, INH, and EMB. The results of the second screen were used as the gold standard and reference for evaluating genetic diagnostic methods. While no multidrug-resistant (MDR) isolates were identified in Saudi Arabia, almost 35% of isolates from Egypt were considered MDR against 3 drugs and 6% MDR against 4 drugs (Table 2). Both numbers are a sharp increase from the original identification of only 6% MDR isolates. Notably, 3.3% of isolates from Saudi Arabia were resistant to streptomycin and ethambutol (Table 2) but did not meet the criteria for MDR according to the WHO guidelines (no double resistance to both isoniazid and rifampicin)^1,32^. Of the 30 isolates from Saudi Arabia, three strains were determined to be resistant to STM by the clinical screening, and this phenotype was confirmed. A 4^th^ strain, SA14, found to be sensitive in the original screening in the KSA, was found to be resistant to STM, and 2 more strains, SA51 and SA28, were found to be partially resistant to INH and EMB, respectively. No strains were found to be resistant to RMP. These results identified three additional drug-resistant strains that were missed by the clinical laboratory.

**Figure 4:**
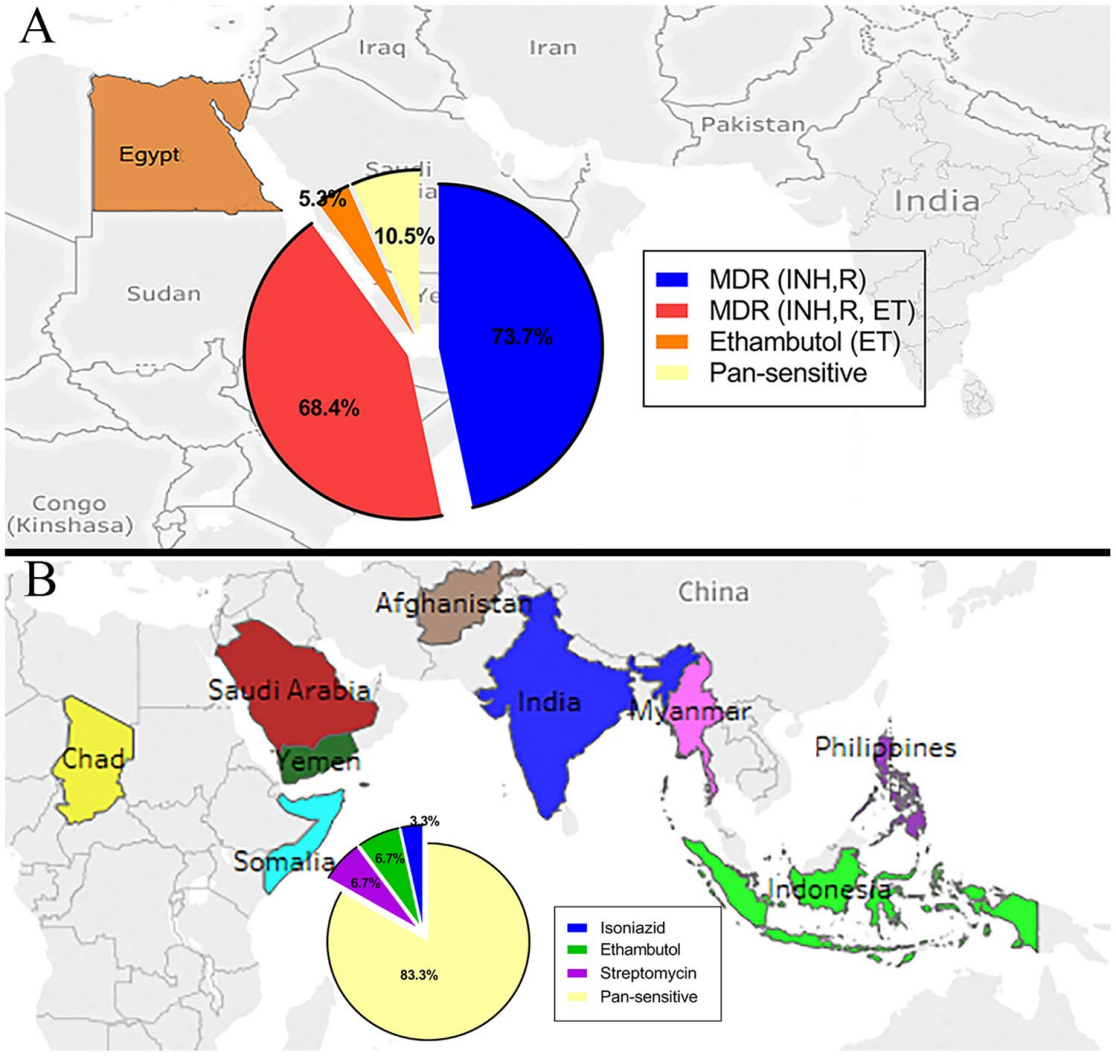
Geographical maps of *M. tuberculosis* drug-resistant isolates examined in this study. (**A**) A map of Egypt with an inset pie chart describing percentages of drug-resistant phenotypes among Egyptian isolates with whole sequenced genomes (N = 19). Note the sum of all percentages >100% because of the high levels of MDR among Egypt isolates. (**B**) A map showing the countries of origin for patients examined in Saudi Arabia. The inset pie chart describing percentages of drug-resistant phenotypes among Saudi Arabian isolates with whole sequenced genomes (N = 30).

In a separate analysis focusing on isolates with whole genome sequences, the country of origin of patients with drug resistant isolates were considered to further characterize potential origin(s) of spread for drug resistance among *M. tuberculosis* isolates (Fig. 4). This analysis revealed that drug-resistant strains did not cluster together (*p* > 0.05); rather, they often grouped with susceptible strains. This was consistent for isolates from both Egypt and Saudi Arabia. Also, drug resistance strains did not cluster together based on patients country of origin (*p* > 0.05). For all screened isolates, the percentages of pansensitive isolates varied significantly between Egypt (10.5%) and Saudi Arabia (83.3%) (Fig. 4). It is noteworthy to mention here that levels of drug resistance were slightly different from those reported in Table 2, most likely because the improved prediction by whole-genome sequencing.

### Molecular determination of drug resistance (DR)

After obtaining accurate DR profiles for the examined *M. tuberculosis* isolates, we attempted to analyze this important phenotype in light of the obtained genome-wide variations, particularly single nucleotide polymorphism (SNPs). For isoniazid, all INH resistant isolates (n = 13, one from Saudi Arabia and 12 from Egypt) were explained by the *fabG1* (*mabA*) C-15T promoter variant or KatG Ser315Asn, both of which have been previously implicated in INH resistance^33,34^. Table S3 represents the variations called for each isolate, including the INH susceptibility testing results. No INH-susceptible isolates had a known resistance-conferring mutation in the queried genes. Two of three isolates from Egypt with no INH susceptibility testing results were likely resistant to INH since the isolates both harbored a KatG Ser315Asn mutation. All RMP resistant isolates harbored a RpoB Ser450Leu variation, which is widely known to confer resistance. Table S4 represents variations for each of the 48 isolates tested for RMP susceptibility. These isolates also harbored a known compensatory mutation in RpoC, Val483Gly, which compensates for the deleterious effects of the Ser450Leu mutation in RpoB^35^. No RMP susceptible isolates (n = 33) harbored a mutation in the RMP resistance-determining region (RRDR). No RMP-susceptible isolates had compensatory mutations in either RpoC or RpoA, agreeing with previous reports^35^.

For ethambutol, all EMB resistant isolates had a mutation previously reported to be associated with EMB resistance. Table S5 represents variations in four genes involved in EMB resistance. Interestingly, several isolates had more than one variation across the four genes, suggesting an increased MIC. However, nine of 28 EMB-susceptible isolates also had these variations, except for the EmbB Gly406Asp; this variation only existed in EMB resistant isolates. Three of the four isolates with no EMB susceptibility testing results also had the EmbB Gly406Asp variation, likely pointing to a resistant phenotype. Of all isolates, only three were phenotypically resistant to PZA, all from Saudi Arabia. Table S6 contains variants called in three genes associated with PZA resistance. Furthermore, none of the three had variations in *pncA* or its promoter region, *rpsA*, or *panD*. This phenomenon is not uncommon and has been reported in several reviews^36–38^. We observed only synonymous substitutions in *pncA* in PZA susceptible isolates (n = 7), which have been reported to not confer resistance^39^; and only one non-synonymous substitution in *rpsA* in a single isolate. Isolates from Egypt did not have PZA susceptibility results, however, 14 of these isolates had a non-synonymous mutation in *pncA* and one had a non-synonymous mutation in *rpsA*. Finally, only 4 isolates from Saudi Arabia and 3 from Egypt were resistant to STM. Only 2 of the 4 STM resistant isolates had a known resistance-conferring mutation in *rpsL* (Lys43Arg) (Table S7). Two resistant isolates from Saudi Arabia did not have a known molecular mechanism to explain the resistant phenotype. One susceptible isolate harbored an *rrs* variation at nucleotide 724, which has not been reported previously. Isolates with no STM susceptibility testing results did not have any variations in the three genes queried.

## Discussion

Tuberculosis, and especially drug-resistant strains of *M. tuberculosis*, represent a major public health threat in developing and underdeveloped countries^32^. With 10 million new TB cases reported each year^40^, regional analyses are of paramount importance to help local health authorities control TB on national levels^41^. Recent analyses of historical and recent genomic data have profiled the nature of tuberculosis disease transmission through global trade, migration, conquests, and even simple travel^42–44^. In the Middle East, most of the reported studies focused on strain lineage distribution and profiling antibiotic sensitivity^10,45,46^. Unfortunately, scarce information is available on the epidemiology of *M. tuberculosis* on the genomic level from the Middle East, compared to other regions around the world^19^. However, several reports have indicated the circulation of DR and MDR in the Middle East^47^, which highlight the need for more thorough genomic analyses. In this report, a comprehensive SNP analysis of *M. tuberculosis* was carried out to identify association with drug resistance phenotypes (TB Profiler and related TBDREAM^48^), and identified poor correlation, most likely because of poor representation of isolates from the Middle East in this database. These results highlight the urgent need for testing of molecular diagnostic methods not only in the Middle East, but also in all understudied regions around the world. It may be that the region of isolation, country of origin, and lineage of the isolate may need to be taken into account when designing molecular diagnostic techniques in the future, and certainly when utilizing them in the field. While sensitivity and specificity information were calculated for RMP, EMB, and STM, the low sample size and <100% specificity or sensitivity reported by TB Profiler did not allow us to make meaningful interpretations of the utility of TB Profiler for the identification of drug-resistant isolates of *M. tuberculosis*. Drug-resistant strains, however, do not appear to be more contagious or spread more widely than drug susceptible strains, unlike earlier analysis of a *M. tuberculosis* Beijing cluster of isolates^49^. Nevertheless, without appropriate diagnosis and treatment of these strains, the risk of spread of drug-resistant strains can only increase. It is noteworthy to mention here that an earlier study on the problems of DR in Saudi Arabia identified a similar pattern for DR to the one reported here with the exception of DR to ethambutol, which was higher in the studied population (4.9%) compared to earlier findings (0.8%)^5^. A more recent study, however, identified DR in up to 8% of isolates within Saudi Arabia^18^.

Phylogenetic analysis of *M. tuberculosis* isolates showed lack of clustering among drug-resistant strains (*p* > 0.05; Fig. 3). In most cases, drug-resistant isolates formed clades with drug-susceptible isolates. Isolates from patients in Egypt formed two distinct clades in lineages 1 and 3, and overall were significantly more closely related to each other than expected by chance. By contrast, isolates from patients in Saudi Arabia were spread amongst all four lineages, and we were unable to reject the null hypothesis that the nationalities of migrant workers were randomly distributed throughout the phylogeny. The relative homogeneity of communities in Egypt compared to the heterogeneity of communities of migrant workers in Saudi Arabia may account for these contrasting patterns. Moreover, lineage 3 was more prevalent among Egypt isolates which contrasted with an earlier study that indicated the prevalence of lineage 4 (Euro-American clade)^13^, most probably due to the analysis of more isolates collected from a more diverse population in the earlier study. Our divergence time estimates indicate that the major clades among closely related Egyptian isolates (lineage 3) have all diverged within the past 78 years compared to >200 years for those isolates (lineage 4) from Saudi Arabia (Fig. 3). Few other lineages exhibit clades with similar divergence dates. Thus, while it is evident that tuberculosis is spread amongst people in Egypt, it is more difficult to determine whether migrant workers contract the disease in Saudi Arabia or in their home countries. The evolutionary diversity of isolates among individuals in Saudi Arabia suggests that this latter scenario likely plays some role. For example, it appears more likely that TB was spread among migrant workers from Somalia while they were living in Saudi Arabia than a scenario where three sister pairs of TB strains were independently introduced into Saudi Arabia from Somalia. By contrast, the four isolates from Chadian individuals included in this analysis belonged to the four different TB lineages. In this case, it is equivocal whether the disease was acquired in Saudi Arabia or introduced from Chad.

Overall, both phenotypic and genome-wide analyses provided more insights into the transmission dynamics of tuberculosis among populations within the Middle East with clearly different demography. Surprisingly, a population (e.g. Saudi Arabia residents) with high exposure to cases of tuberculosis with DR and MDR-TB, appear to have less of a problem of DR tuberculosis based on our analysis and others^5^. On the other hand, a homogenous population structure (mainly Egyptians) had an increasing problem of MDR^17^, an important highlight to the problem of adherence to drug therapy and a suitable target for the STOP program by the WHO. More importantly, considerations need to be given to genomic analysis of more *M. tuberculosis* isolates from different countries and to the prevalent lineage as well as drug-resistant genotypes by public health authorities before selection of a suitable diagnostic or drug regiment for a country within the Middle East.

## Methods

### Ethics statement and isolation of *M. tuberculosis*

Approval to use patient samples was obtained from the Institutional Review Board of Kaser Elini Faculty of Medicine, Cairo University as well as the Ministry of Health of the Kingdom of Saudi Arabia, and the Research Ethics committee, Faculty of Medicine, King Abdulaziz University. All isolates were de-identified to maintain patient privacy while information related to the patient gender, nationality, age and city of residency were kept to be used for the analysis of transmission dynamics. Approval to use de-identified clinical isolates of *M. tuberculosis* was also obtained from the University of Wisconsin-Madison’s Institutional Review Board. All methods conducted in this research adhered to the guidelines and regulations approved by the Office of Biological Safety at the University of Wisconsin-Madison.

All clinical isolates of *M. tuberculosis* were obtained from early morning sputum specimens. Sputum samples were digested and decontaminated by the ready to use N- acetyl- l- cysteine (NALC) 3% NaOH method (Nac-PACTMEA3, Alpha Tec, Inc, Vancouver, Washington, USA) and concentrated by centrifugation at 3,000 rpm for 20 minutes, as described by the manufacturer^50^. The resulting pellet was resuspended and used in part for detection of *M. tuberculosis* complex (MTBC) isolates by smear microscopy using fluorochrome stain (Fluo-RAL-Auramine staining kit for Mycobacteria Detection). The remainder of the resuspended pellet was used for culturing in the VersaTREK fluid culture system and for direct MTBC detection by Gene Xpert PCR. All clinical isolates were grown on Löwenstein–Jensen medium slants. Single colonies from each isolate were sub-cultured on Middlebrook 7H10 agar (BD, Franklin Lakes, NJ) supplemented with 0.5% glycerol and 10% ADC (2% glucose, 5% bovine serum albumin, fraction V, and 0.85% NaCl), and incubated at 37 °C under biosafety level 3 (BSL3) laboratory conditions. Single colonies were grown in Middlebrook 7H9 broth (BD Difco) supplemented with 0.5% glycerol and 10% ADC for genomic DNA extraction and drug resistance testing.

### Phenotypic analysis

VersaTREK 528 (TREK Diagnostic Systems, Inc. Westlake, OH) was used to determine phenotypic drug resistance in the place of isolation (KAUH) following the manufacturer’s instructions. Inoculated cultures containing rifampicin (30 µg/ml), isoniazid (3 µg/ml or 12 µg/ml), ethambutol (150 µg/ml or 240 µg/ml), streptomycin (30 µg/ml or 120 µg/ml), or pyrazinamide (300 µg/ml) and were monitored for a positive signal for up to 42 days^51^. For further confirmation following sequence analysis, antibiotic sensitivity of selected isolates was determined using standard, semi-quantitative *in vitro* susceptibility testing. In this assay, Middlebrook 7H10 agar plates were embedded with BBL Sensi-Disc Susceptibility Test Discs (BD Biosciences, MD) to reach concentrations of 0.2 µg/ml INH, 5 µg/ml RMP, 10 µg/ml STM, 5 µg/ml EMB, or left free of antibiotic as a positive control. Plates were inoculated with the 50 µl of each isolate, directly or serially diluted from early log phase cultures grown in Middlebrook 7H9 broth to an OD_600_ of 0.1–0.2 and incubated at 37 °C for 21–28 days.

Finally, the GeneXpert Dx automated system (Cepheid AB, Solana, Sweden) was used to detect MTBC and rifampicin resistant genotypes at the KAUH following the manufacturer’s instructions as previously described^52–55^. GenoType MTBDRplus (Hain Lifescience GmbH, Nerhren, Germany) was also used to detect MTBC and rifampicin as well as isoniazid resistant genotypes^56^. GenoType Mycobacterium CM was used to identify nontuberculous mycobacteria (NTM) including *M. avium* ssp., *M. intracellulare*, and *M. chelonae*^57^.

### Genotyping of *M. tuberculosis* isolates

Genomic DNA was extracted from *M. tuberculosis* cultures as previously described^58^. For mycobacterial interspersed repetitive units (MIRU), 12 previously identified loci were examined using published primer sets and PCR reaction mixes^22^. Amplified loci were run on 2% NuSeive agarose gels alongside 20 and 100 bp ladders. Fragment size data and antibiotic resistance information, if applicable, were entered into MIRU-VNTR plus^25^ as well as TBinsight^26^ for analysis to determine the lineages of all isolates. The MIRU-based lineage data was used to select representative samples from each lineage for whole genome sequencing.

Genomic DNA samples were prepared at the University of Wisconsin-Madison Biotechnology Center and sequenced on Illumina HiSeq2500 or MiSeq platforms. Adapters were trimmed from the raw reads using Trimmomatic (v0.36)^59^ and subsequently aligned using Bowtie2 (v2.2.4)^27^ using default parameters to the *M. tuberculosis* H37Rv strain (GenBank accession NC_000962.3). Using SAMtools (v1.3.1)^60^, the BAM files were sorted and the mpileup function was used to create an mpileup file; a minimum mapping quality of 20 was set, otherwise default parameters were used. Variants were called with VarScan2 (v2.3) mpileup2cns^28^ with a minimum variant quality of 20, a minimum depth of 10, and the variants parameter was flagged to only output variants; default parameters were used otherwise. All variant types (SNPs, insertions, and deletions) were considered in drug resistance association. A total of 20 SNPs shared between 3–5 different isolates including 10 involved in drug resistance were selected for confirmation by Sanger sequencing. Primers used for sequencing are shown in Table S2.

### Genotypic-Phenotypic Association

For each drug, known resistance-conferring genes and variants were identified through a literature search and queried across all genomes (Tables S3–7. For a complete list of genes and genome positions queried in the whole-genome sequencing data, see Table S8. Isolates were considered “explained” if the genotype matched the phenotype (e.g. KatG Ser315Thr associated with INH resistance or no variation in codon 315 in INH susceptible); otherwise, the isolates were considered “unexplained” if the genotype did not match the phenotype (e.g. resistance to RIF detected by DST but no mutation was detected in the RRDR).

### Phylogenetic analysis

For phylogenetic analysis, the consensus sequence of each isolate was extracted from CLCBio Genomics Workbench 8 after reference assembly. The reference strain *M. tuberculosis* H37Rv (NC_000962.3) and several other available *M. tuberculosis* genomes representing various lineages were included in a phylogenetic tree created using ParSNP, part of the Harvest genome software developed by the University of Maryland Center for Bioinformatics and Computational Biology. In general, ~ 230 genomes were downloaded from GenBank representing isolates from various regions throughout the world, and then aligned to sequenced genomes from KAUH isolates. FastTree 2 within ParSNP was used to build a phylogenetic tree with an approximately-maximum-likelihood method taking into consideration all SNPs, indels, and structural differences found in the genomes^61^. We then developed a chronogram using an alignment of 17,348 SNPs using Beast v.2.4.7^62^ in order to estimate divergence times among the isolates. The best fitting model of molecular evolution was identified with jModelTest v2.1.4^63^. We used three age priors with a loose uniform distribution to establish hard bounds for the backbone of the chronogram, based on the confidence intervals surrounding dates obtained in the more comprehensively sampled analyses of Bos *et al*.^31^. These included a prior on the most recent common ancestor (MRCA) of lineages 1–4 (2.7–5.1 Kya), lineages 2–4 (1.7–3.4 Kya), and lineages 2–3 (1.6–3.1 Kya). We ran two separate analyses, one where a strict clock was enforced and a second analysis where rates were allowed to vary among all branches. We then compared the likelihood of both analyses using likelihood ratio tests. Analyses were run for 200 million generations, with stabilization and convergence assessed using Tracer v1.6^64^.

For *In silico* spoligotyping and drug resistance prediction, assembled whole genome sequences were uploaded to the CASTB website (http://castb.ri.ncgm.go.jp/CASTB/) where they were analyzed for spoligotype based on SPOLDB4^65^. For genetic prediction of drug resistance, raw fastq files were uploaded to the TB Profiler website which provided information on mutations correlated with drug resistance and mutations located in genes known to be correlated with drug resistance. We also tested if a significant phylogenetic signal existed for drug resistance and for patient’s age, gender, or nationality in order to determine if we could identify modes or locations of disease transmission, and how these may differ in Egypt and Saudi Arabia. Isolates were placed into pseudo-communities according to these traits, and we tested whether the average phylogenetic distance among isolates within these communities was greater or smaller than expected compared a null model. These analyses were conducted using the ses.mpd function in the R package picante^66^.

### Statistical analysis

All association studies between genotype, genome wide studies, and phenotypes of *M. tuberculosis* isolates were conducted using statistical modules described with each conducted analysis, as detailed above. All other simple statistical analyses of lineage distributions were calculated using statistical packages present in Microsoft Excel and GraphPad 5.0 softwares. A *p* ≤ *0.05* was considered signficant when tests of phylogenetic signal were used to determine whether drug-resistant isolates were more closely related to each other than expected by chance, or whether patients were significantly clustered accroding to their age, nationality, or gender. These analyses were conducted using the ses.mpd function in picante^66^.

## Supplementary information

Supplemtal tables

## References

[1] Zumla A, George A, Sharma V, Herbert N, Baroness Masham of I (2013). WHO’s 2013 global report on tuberculosis: successes, threats, and opportunities. Lancet.

[2] Verdugo D (2015). Epidemiologic Correlates of Pyrazinamide-Resistant *Mycobacterium tuberculosis* in New York City. Antimicrob. Agents Chemother..

[3] Glaziou P, Sismanidis C, Floyd K, Raviglione M (2014). Global epidemiology of tuberculosis. Cold Spring Harb Perspect Med.

[4] Sulis G (2016). Recent developments in the diagnosis and management of tuberculosis. NPJ Prim Care Respir Med.

[5] Al-Hajoj S (2013). Epidemiology of antituberculosis drug resistance in Saudi Arabia: findings of the first national survey. Antimicrob Agents Chemother.

[6] Al-Mazrou YY, Khoja TAM, Aziz KMS, Salem AM (1997). High proportion of multi-drug resistant *Mycobacterium tuberculosis* in Saudi Arabia. Scand.J.Infect.Dis..

[7] Morcos W (2008). Drug-resistant tuberculosis in Egyptian children using Etest. Minerva pediatrica.

[8] Abbadi SH, Sameaa GA, Morlock G, Cooksey RC (2009). Molecular identification of mutations associated with anti-tuberculosis drug resistance among strains of *Mycobacterium tuberculosis*. International journal of infectious diseases: IJID: official publication of the International Society for Infectious Diseases.

[9] Young F, Critchley J, Johnstone L, Unwin N (2009). A review of co-morbidity between infectious and chronic disease in Sub Saharan Africa: TB and Diabetes Mellitus, HIV and Metabolic Syndrome, and the impact of globalization. Globalization and Health.

[10] Gleason JA (2012). Tuberculosis trends in the Kingdom of Saudi Arabia, 2005 to 2009. Ann Epidemiol.

[11] Dalla Costa ER (2015). Multidrug resistant *Mycobacterium tuberculosis* of the Latin American Mediteranean lineage wrongly identified as Mycobacterium pinnipedii (ST863) causing active tuberculosis in south Brazil. J Clin Microbiol.

[12] Somily AM (2014). Changing epidemiology of tuberculosis detected by an 8-year retrospective laboratory study in a tertiary teaching hospital in central Saudi Arabia. Saudi Med J.

[13] Diab HM (2016). First insight into the genetic population structure of *Mycobacterium tuberculosis* isolated from pulmonary tuberculosis patients in Egypt. Tuberculosis.

[14] Ellis ME, Al-Hajjar S, Bokhari H, Qadri SMH (1996). High proportion of multi-drug resistant *Mycobacterium tuberculosis* in Saudi Arabia. Scand.J.Infect.Dis..

[15] Al Hajoj SAM (2007). First Insight into the Population Structure of *Mycobacterium tuberculosis* in Saudi Arabia. Journal of Clinical Microbiology.

[16] Al Tawfiq JA, Al Muraikhy AA, Abed MS (2005). Susceptibility pattern and epidemiology of *Mycobacterium tuberculosis* in a Saudi Arabian hospital - A 15-year study from 1989 to 2003. Chest..

[17] Abbadi S, El Hadidy G, Gomaa N, Cooksey R (2009). Strain differentiation of *Mycobacterium tuberculosis* complex isolated from sputum of pulmonary tuberculosis patients. International journal of infectious diseases: IJID: official publication of the International Society for Infectious Diseases.

[18] Coll F (2018). Genome-wide analysis of multi- and extensively drug-resistant *Mycobacterium tuberculosis*. Nat Genet.

[19] Manson AL (2017). Genomic analysis of globally diverse *Mycobacterium tuberculosis* strains provides insights into the emergence and spread of multidrug resistance. Nature Genetics.

[20] Wilder-Smith A, Foo W, Earnest A, Paton NI (2005). High risk of *Mycobacterium tuberculosis* infection during the Hajj pilgrimage. Tropical Medicine & International Health.

[21] Rahman J, Thu M, Arshad N, Van der Putten M (2017). Mass Gatherings and Public Health: Case Studies from the Hajj to Mecca. Ann Glob Health.

[22] Supply P (2006). Proposal for standardization of optimized mycobacterial interspersed repetitive unit-variable-number tandem repeat typing of *Mycobacterium tuberculosis*. J Clin Microbiol.

[23] Gagneux S (2006). Variable host-pathogen compatibility in *Mycobacterium tuberculosis*. Proceedings of the National Academy of Sciences of the United States of America..

[24] Al-Hajoj SAM (2007). First insight into the population structure of *Mycobacterium tuberculosis* in Saudi Arabia. Journal of Clinical Microbiology.

[25] Allix-Béguec C, Harmsen D, Weniger T, Supply P, Niemann S (2008). Evaluation and Strategy for Use of MIRU-VNTRplus, a Multifunctional Database for Online Analysis of Genotyping Data and Phylogenetic Identification of *Mycobacterium tuberculosis* Complex Isolates. Journal of Clinical Microbiology.

[26] Shabbeer A (2012). TB-Lineage: an online tool for classification and analysis of strains of *Mycobacterium tuberculosis* complex. Infection, genetics and evolution: journal of molecular epidemiology and evolutionary genetics in infectious diseases.

[27] Langmead B, Salzberg SL (2012). Fast gapped-read alignment with Bowtie 2. Nat Methods.

[28] Koboldt DC (2012). VarScan 2: somatic mutation and copy number alteration discovery in cancer by exome sequencing. Genome Res.

[29] Guerra-Assuncao JA (2015). Recurrence due to Relapse or Reinfection With *Mycobacterium tuberculosis*: A Whole-Genome Sequencing Approach in a Large, Population-Based Cohort With a High HIV Infection Prevalence and Active Follow-up. The Journal of Infectious Disease.

[30] Coll F (2012). SpolPred: rapid and accurate prediction of *Mycobacterium tuberculosis* spoligotypes from short genomic sequences. Bioinformatics.

[31] Bos KI (2014). Pre-Columbian mycobacterial genomes reveal seals as a source of New World human tuberculosis. Nature.

[32] Anon. Global tuberculosis report 2017. (World Health Organization., Geneva, 2017).

[33] Torres JN (2015). Novel katG mutations causing isoniazid resistance in clinical M. tuberculosis isolates. Emerg Microbes Infect.

[34] Tseng ST, Tai CH, Li CR, Lin CF, Shi ZY (2015). The mutations of katG and inhA genes of isoniazid-resistant *Mycobacterium tuberculosis* isolates in Taiwan. J Microbiol Immunol Infect.

[35] Comas I (2011). Whole-genome sequencing of rifampicin-resistant *Mycobacterium tuberculosis* strains identifies compensatory mutations in RNA polymerase genes. Nat Genet.

[36] Ramirez-Busby SM, Valafar F (2015). Systematic review of mutations in pyrazinamidase associated with pyrazinamide resistance in *Mycobacterium tuberculosis* clinical isolates. Antimicrob Agents Chemother.

[37] Whitfield MG (2015). A Global Perspective on Pyrazinamide Resistance: Systematic Review and Meta-Analysis. PLoS One.

[38] Yadon AN (2017). A comprehensive characterization of PncA polymorphisms that confer resistance to pyrazinamide. Nat Commun.

[39] Scorpio A, Zhang Y (1996). Mutations in pncA, a gene encoding pyrazinamidase/nicotinamidase, cause resistance to the antituberculous drug pyrazinamide in tubercle bacillus. Nat Med.

[40] Dye C, Williams BG (2010). The population dynamics and control of tuberculosis. Science..

[41] Pepperell C (2010). Bacterial Genetic Signatures of Human Social Phenomena among M-tuberculosis from an Aboriginal Canadian Population. Molecular Biology and Evolution.

[42] Pepperell CS (2011). Dispersal of *Mycobacterium tuberculosis* via the Canadian fur trade. Proc Natl Acad Sci USA.

[43] Gagneux S (2012). Host-pathogen coevolution in human tuberculosis. Philos Trans R Soc Lond B Biol Sci.

[44] Casper C (1996). The transcontinental transmission of tuberculosis: A molecular epidemiological assessment. American.Journal.of.Public.Health..

[45] Bassili A (2010). Estimating tuberculosis case detection rate in resource-limited countries: a capture-recapture study in Egypt. Int.J Tuberc.Lung Dis..

[46] Sharaf Eldin GS (2011). Tuberculosis in Sudan: a study of *Mycobacterium tuberculosis* strain genotype and susceptibility to anti-tuberculosis drugs. BMC Infect Dis.

[47] Shibl A, Senok A, Memish Z (2012). Infectious diseases in the Arabian Peninsula and Egypt. Clinical microbiology and infection: the official publication of the European Society of Clinical Microbiology and Infectious Diseases.

[48] Coll F (2015). Rapid determination of anti-tuberculosis drug resistance from whole-genome sequences. Genome Med.

[49] Huyen MNT (2013). Tuberculosis Relapse in Vietnam Is Significantly Associated With *Mycobacterium tuberculosis* Beijing Genotype Infections. Journal of Infectious Diseases.

[50] Moure R (2011). Rapid detection of *Mycobacterium tuberculosis* complex and rifampin resistance in smear-negative clinical samples by use of an integrated real-time PCR method. J Clin Microbiol.

[51] Marlowe EM (2011). Evaluation of the Cepheid Xpert MTB/RIF assay for direct detection of *Mycobacterium tuberculosis* complex in respiratory specimens. J Clin Microbiol.

[52] Boehme CC (2010). Rapid molecular detection of tuberculosis and rifampin resistance. N.Engl.J Med..

[53] Rattan A, Kalia A, Ahmad N (1998). Multidrug-resistant *Mycobacterium tuberculosis*: molecular perspectives. Emerg Infect Dis.

[54] Zeka AN, Tasbakan S, Cavusoglu C (2011). Evaluation of the GeneXpert MTB/RIF assay for rapid diagnosis of tuberculosis and detection of rifampin resistance in pulmonary and extrapulmonary specimens. J Clin Microbiol.

[55] Omrani AS (2014). GeneXpert MTB/RIF Testing in the Management of Patients with Active Tuberculosis; A Real Life Experience from Saudi Arabia. Infect Chemother.

[56] Crudu V (2012). First evaluation of an improved assay for molecular genetic detection of tuberculosis as well as rifampin and isoniazid resistances. J Clin Microbiol.

[57] Hillemann D, Rusch-Gerdes S, Boehme C, Richter E (2011). Rapid molecular detection of extrapulmonary tuberculosis by the automated GeneXpert MTB/RIF system. J Clin Microbiol.

[58] Hsu C-Y, Wu C-W, Talaat AM (2011). Genome-wide sequence variations among Mycobacterium avium subspecies paratuberculosis isolates: A better understanding of Johne’s disease transmission dynamics. Front Microbiol..

[59] Bolger AM, Lohse M, Usadel B (2014). Trimmomatic: a flexible trimmer for Illumina sequence data. Bioinformatics.

[60] Li H (2009). The Sequence Alignment/Map format and SAMtools. Bioinformatics..

[61] Treangen TJ, Ondov BD, Koren S, Phillippy AM (2014). The Harvest suite for rapid core-genome alignment and visualization of thousands of intraspecific microbial genomes. Genome Biol.

[62] Bouckaert R (2014). BEAST 2: a software platform for Bayesian evolutionary analysis. PLoS Comput Biol.

[63] Darriba D, Taboada GL, Doallo R, Posada D (2012). jModelTest 2: more models, new heuristics and parallel computing. Nat Methods.

[64] Tracer v1.6 (http://tree.bio.ed.ac.uk/software/tracer/ 2014).

[65] Brudey K (2006). *Mycobacterium tuberculosis* complex genetic diversity: mining the fourth international spoligotyping database (SpolDB4) for classification, population genetics and epidemiology. BMC Microbiol.

[66] Kembel SW (2010). Picante: R tools for integrating phylogenies and ecology. Bioinformatics.

