# Publisher Correction: Genomic analysis of the emergence of drug-resistant strains of *Mycobacterium tuberculosis* in the Middle East

**DOI:** 10.1038/s41598-019-55790-8

**Published:** 2019-12-27

**Authors:** Essam J. Alyamani, Sarah A. Marcus, Sarah M. Ramirez-Busby, Chungyi Hansen, Julien Rashid, Amani El-kholy, Daniel Spalink, Faramarz Valafar, Hussein A. Almehdar, Asif A. Jiman-Fatani, Mohamed A. Khiyami, Adel M. Talaat

**Affiliations:** 1 National Center for Biotechnology, King Abdulaziz City for Science and Technology, Riyadh, Saudi Arabia; 2 Department of Pathobiological Sciences, University of Wisconsin-Madison, Madison, WI USA; 3 Laboratory for Pathogenesis of Clinical Drug Resistance and Persistence, Biomedical Informatics Research Center, San Diego State University, San Diego, CA USA; 4 Clinical Pathology Department, Faculty of Medicine Cairo University, Cairo, Egypt; 5 Department of Ecosystem Science and Management, Texas A&M University, College Station, TX USA; 6 Department of Biology, Faculty of Science, King Abdulaziz University, Jeddah, Saudi Arabia; 7 Department of Medical Microbiology and Parasitology, Faculty of Medicine, King Abdulaziz University, Jeddah, Saudi Arabia

Correction to: *Scientific Reports* 10.1038/s41598-019-41162-9, published online 14 March 2019

In the original version of this Article, there were errors in Affiliations of authors Hussein A. Almehdar and Asif A. Jiman-Fatani which were incorrectly listed as ‘Department of Microbiology and Medical Parasitology, Faculty of Medicine, King Abdulaziz University, Jeddah, Saudi Arabia’ and ‘Department of Biology, Faculty of Science, King Abdulaziz University, Jeddah, Saudi Arabia’ respectively.

The correct affiliations are listed below.

Affiliation of Hussein A. Almehdar:

Department of Biology, Faculty of Science, King Abdulaziz University, Jeddah, Saudi Arabia.

Affiliation of Asif A. Jiman-Fatani:

Department of Medical Microbiology and Parasitology, Faculty of Medicine, King Abdulaziz University, Jeddah, Saudi Arabia.

These errors have now been corrected in the PDF and HTML versions of the Article.

In the original version of this Article, the author Asif A. Jiman-Fatani was incorrectly indexed. This error has now been corrected.

